# Establishing a Bridge Between Supervisor’s Perceived Organizational Support and Salesperson’s Career Initiative

**DOI:** 10.3390/bs15050617

**Published:** 2025-05-01

**Authors:** Yiran Gai, Guicheng Shi, Yu Liu, Zhitao Chen

**Affiliations:** 1 School of Business, Macau University of Science and Technology, Avenida Wai Long, Taipa, Macau 999078, China; gaiyiran@foxmail.com (Y.G.); 2009853zbm30001@student.must.edu.mo (Z.C.); 2 School of Management, Zhejiang University, Hangzhou 310058, China; 22320175@zju.edu.cn

**Keywords:** supervisor’s perceived organizational support, group-inclusive climate, felt obligation, career initiative, core self-evaluation

## Abstract

The proactive behavior of front-line salespeople plays a crucial role in generating positive organizational outcomes. As the managers who interact most frequently with front-line employees, front-line managers’ perception of organizational support is pivotal in fostering the development of career initiative among these employees. Grounded in self-determination and social exchange theories, this study investigates whether the organizational support perceived by front-line managers influences employee initiative behavior. Meanwhile, core self-evaluation is introduced to explore how personality traits of sales personnel may influence their perception of the external environment and the formation of intrinsic motivation. This study utilized a questionnaire survey method to collect data from 50 front-line team leaders and their 299 corresponding employees across multiple cities in China, conducted over three rounds. Following the collection of the paired questionnaires, Mplus 8.0 was employed to perform reliability and validity analyses, correlation analysis, and hypothesis testing on the data. The final results revealed that supervisor’s perceived organizational support positively influences a group-inclusive climate; a group-inclusive climate can foster felt obligation and salesperson career initiative. Supervisors’ perceived organizational support enhances salesperson career initiative by making salespeople experience group-inclusive climate and develop felt obligation. Moreover, core self-evaluation significantly moderates the positive impact of the inclusive climate on these outcomes. By adopting the perspective of front-line supervisors, this research identifies an effective pathway from supervisor perception to employee behavior, elucidates the antecedents of front-line salespeople’s initiative, and reassesses the critical role of front-line supervisors within organizations.

## 1. Introduction

The responsibilities of salespeople have fundamentally transformed due to their pivotal role in executing corporate strategies. The traditional model, which evaluates performance primarily through revenue and profit, is no longer sufficient (Elsaied, 2025; Forkmann et al., 2022; Kraft et al., 2019). In response to growing uncertainty and intense competition, companies now expect employees to take more proactive initiatives (Nwanzu & Babalola, 2024). Previous organizational-level research has predominantly focused on motivating employees’ proactive behaviors by emphasizing the organizational climate (Kang et al., 2016), human resource development (Sim et al., 2024; Van Lancker et al., 2022), and leadership styles (Mäkelä et al., 2024). Nevertheless, the distinct role of front-line managers within organizations has received relatively little attention (Zhao et al., 2024). Given that front-line employees interact more frequently with these managers and view them as organizational representatives (Eisenberger et al., 2014), enhancing employee proactivity may require greater involvement from front-line managers.

The business community has taken the lead over academia in supporting front-line managers through management practices. For instance, the Chinese supermarket chain Pang Donglai empowers its front-line supervisors, thereby enabling sales staff to exhibit more proactive behaviors. This initiative has not only significantly boosted revenue and enhanced customer reputation but also established Pang Donglai as one of China’s leading private supermarkets (Qian, 2024 in Chinese). This case highlights the untapped potential of front-line managers who not only influence employee behavior but also directly enhance organizational performance (Kou et al., 2022; Chang et al., 2020). Additionally, research indicates that front-line managers play a critical role in determining the job satisfaction and performance of front-line employees (Karltun et al., 2023; Huo et al., 2020), serving as the crucial link between top management and the interests of employees (Townsend & Kellner, 2015).

Although front-line managers play a crucial role, academic research on motivating them and providing positive feedback to front-line employees remains underdeveloped (Zhao et al., 2024; Doldor et al., 2019). This is significant because only by adopting a positive attitude can front-line managers create an environment that fosters proactive behavior among employees. However, front-line managers may be reluctant to assist employees, particularly in a negative organizational climate, which can undermine collaboration, focus, and creativity (Karltun et al., 2023). Adequate organizational support is vital for front-line managers to form positive expectations, alleviate uncertainty-related pressure (Tan et al., 2024) and enhance interactions with employees. In summary, it is imperative to consider front-line managers’ perceptions of the organizational environment. They require organizational support not only to ensure positive social exchanges (Eisenberger et al., 2014) but also to encourage a greater investment of time and effort in front-line operations (Aldabbas et al., 2023), ultimately enhancing the performance of front-line employees.

This study investigates the reasons for front-line managers’ perceptions of organizational support based on three key considerations. First, unlike prior research focusing on supervisors’ personality traits or leadership styles, this study highlights organizational support as a situational factor that organizations can actively improve and adjust (Ding et al., 2022) This support directly influences employees’ attitudes and behaviors by providing practical opportunities for front-line managers (Op De Beeck et al., 2018). Second, career initiative requires emotional commitment and autonomous motivation (Ayed et al., 2024), both of which are closely linked to perceived organizational support (Eisenberger et al., 2020). Front-line employees’ perception of organizational support is mediated through supervisors: when supervisors perceive support, they reciprocate by fostering positive leader–member exchanges (LMX) and offering emotional and resource support to subordinates, enabling front-line employees to experience organizational support (Eisenberger et al., 2014). Therefore, we argue that supervisor’s perceived organizational support (SPOS) serves as a critical driver of career initiatives among front-line personnel. Thirdly, analyzing the antecedents and mechanisms of career initiative among front-line salespeople enhances the logical framework of proactive behavior research.

While some scholars have noted that SPOS can foster employees’ innovative behavior (Pan et al., 2012) or reduce withdrawal behavior (Eisenberger et al., 2014), these studies do not explicitly elucidate the underlying motivations driving such employee behaviors. To address this gap, we incorporate self-determination theory (SDT), which posits that external environmental factors can facilitate intrinsic motivation, enabling individuals to autonomously choose their behaviors. According to SDT, both the environment and intrinsic motivation are critical determinants of behavior (Deci et al., 2017).

We argue that when front-line managers perceive organizational support, they can foster an inclusive environment through their behaviors, promoting mutual respect and harmonious interactions among employees. This, in turn, creates the necessary conditions for stimulating autonomous motivation (Shore et al, 2011; Randel et al., 2018). Although LMX theory partially explains this phenomenon, the concept of inclusiveness provides a more comprehensive description of the overall organizational environment. Furthermore, the transformation of supervisors’ perceptions directly influences changes in the team environment (Carmeli et al., 2010), as an inclusive climate encompasses team-level interactions rather than being limited to dyadic relationships between supervisors and individual employees (Shore & Chung, 2024).

On the other hand, front-line sales personnel who perceive support from their supervisors and an inclusive team environment develop a strong sense of obligation toward the organization, becoming “responsible citizens” (Cheng et al., 2022). Felt obligation serves as a key motivational factor driving proactive behaviors (Eisenberger et al., 2014). Although variables such as psychological safety can also encourage such behaviors, felt obligation uniquely influences front-line sales personnel due to their significant autonomy and limited direct supervision (Lyngdoh et al., 2023). A robust sense of responsibility is crucial for balancing freedom with mission fulfillment (Thompson et al., 2020), fostering self-driven proactivity (Ayed et al., 2024). Therefore, we emphasize inclusive climate and felt obligation as critical mechanisms explaining how perceived organizational support from supervisors translates into proactive behaviors among front-line sales personnel.

Furthermore, it is crucial to recognize that external environmental factors and the personal characteristics of salespeople may jointly influence the generation of proactive behavior motivation (Zhao et al., 2024). While positive social exchange is a universal human trait, individuals vary in their acceptance of the balance between giving and receiving (Cropanzano & Mitchell, 2005). This dimension has received limited attention in studies of autonomous motivation. We thus incorporate core self-evaluation (CSE) as a pivotal factor shaping salespeople’s positive choices. High CSE strengthens self-efficacy and fosters self-determination, whereas low CSE may impede proactivity even with managerial support due to inadequate self-esteem and confidence (Li & Ding, 2022). Based on this discussion, this study proposes the following research questions (RQ):

RQ1: How does the organizational support perceived by front-line managers influence the career initiative of front-line salespeople?

RQ2: Do group-inclusive climate and felt obligation mediate the relationship between SPOS and salesperson’s career initiative?

RQ3: Does salesperson’s CSE moderate the relationships between group-inclusive climate and felt obligation, as well as between group-inclusive climate and salesperson’s career initiative?

To address the above questions, we propose a theoretical framework that elucidates how SPOS influences salespeople’s career initiative. This study makes three key contributions: First, we extend the antecedents of career initiative among front-line salespeople and delineate a pathway from supervisors’ perceptions to career initiative, with practical implications for management practice. Second, by integrating self-determination theory and social exchange theory, we clarify how supervisors’ perceptions indirectly foster career initiative through creating an inclusive team climate and salespeople’s felt obligation. Third, we investigate the moderating role of core self-evaluation (CSE) in shaping career initiative, offering insights into why some employees exhibit greater proactivity than others despite comparable levels of organizational support.

The structure of this article is as follows. The first part reviews the literature about SPOS, group-inclusive climate, felt obligation, career initiative, and core self-evaluation, and it formulates the hypotheses for this study. The second part provides a detailed overview of the research methods. In the third part, the analysis and results are thoroughly presented. The fourth part provides a detailed discussion of the study findings, and the final part provides a summary of the conclusions, addresses the limitations, and outlines directions for future research.

## 2. Literature Review and Hypothesis Development

### 2.1. Supervisor’s Perceived Organizational Support and Group-Inclusive Climate

As a team manager, the supervisor functions both as an employee and a leader (Eisenberger et al., 2002). SPOS reflects a manager’s perception of whether organizational members or superior leaders value their contributions to the organization’s development and care about their well-being (Eisenberger et al., 2002). Research shows that SPOS enables employees to perceive organizational support through leader–member exchanges (Eisenberger et al., 2014). When managers feel fully supported by the organization, they are more likely to provide developmental feedback to motivate their employees (Zhao et al., 2024). prompting team members to recognize changes in their manager’s behavior and develop a strong sense of organizational support (Eisenberger et al., 2014). Subsequent research has shown that SPOS can result in a series of changes in the sense of support, including supervisor support, co-worker support, and the supportive atmosphere of the group as a whole (Puah et al., 2016). In such environments, team members exhibit mutual respect, offer reciprocal assistance, and foster a strong sense of belonging.

Organizational support is closely tied to the creation of an inclusive environment (Cancela et al., 2022; Ashikali & Groeneveld, 2015). Stamper and Masterson (2002) demonstrated that perceptions of inclusion parallel perceptions of organizational support, both enhancing employees’ sense of belonging and recognition. Nembhard and Edmondson (2006) defined inclusive leaders as those who attend to employee needs, listen to their views, encourage them, and recognize their contributions. Carmeli et al. (2010) expanded this concept by describing inclusivity as the support, openness, and approachability demonstrated in team interactions. These definitions indicate that supportive behaviors from both supervisors and team members foster an inclusive work environment. Based on reciprocity theory, when managers perceive strong organizational support, they exhibit prosocial behaviors benefiting their teams (Kurtessis et al., 2017), thereby extending greater support to team members. Employees then emulate this behavior, promoting positive social exchanges. Through mutual reinforcement, employees and managers collaboratively create a supportive and inclusive workplace.

**Hypothesis** **1.** *SPOS has a positive impact on a group-inclusive climate.*

### 2.2. Group-Inclusive Climate, Salesperson’s Felt Obligation, and Career Initiative

The self-determination theory posits that employees select behaviors driven by the satisfaction of their internal psychological needs in response to changes in the external environment (Deci et al., 1989). This connects intrinsic motivation to the interplay between the environment and individual actions. Felt obligation serves as a critical internal factor that fosters positive employee behavior, motivating employees to prioritize the organization’s welfare and contribute to its goals (Eisenberger et al., 2001; Malhotra et al., 2022). For front-line sales staff, voluntary commitment plays a pivotal role in enhancing service performance, given their relatively flexible work schedules and locations (Malhotra et al., 2022). This flexibility enables salespeople to make more autonomous decisions compared to employees in other departments, constituting a fundamental aspect of their service role (Boshoff & Allen, 2000). Felt obligation ensures that salespeople fulfill their responsibilities even in a flexible work setting and may inspire them to exceed expectations (Settoon et al., 1996).

Pan et al. (2012) and Vadera et al. (2013) developed a framework demonstrating that perceived organizational support, encompassing both supervisor and colleague support, enhances employees’ felt obligation. Building on this foundation, we examine how a group-inclusive climate, fostered by such support, strengthens front-line salespeople’s felt obligation. A group-inclusive climate constitutes part of the external environment perceived by employees (Carmeli et al., 2010), promoting organizational openness and enhancing team identity among salespeople. When inclusion is perceived, salespeople engage in social exchange processes, believing that “goodwill will be rewarded in the future” (Mossholder et al., 2005). This belief motivates them to feel obligated to assist the organization in achieving its goals and to anticipate greater rewards for increased effort (Kurtessis et al., 2017).

**Hypothesis** **2.** *A group-inclusive climate has a positive impact on a salesperson’s felt obligation.*

A group-inclusive climate empowers salespeople to engage in proactive behaviors that enhance team efficiency (Carmeli et al., 2010), such as redefining organizational goals to pursue more challenging work (Hacker, 1985). However, this necessitates adequate organizational support to foster psychological security, self-efficacy, and career commitment among employees (Norton et al., 2014; Carmeli et al., 2010; Choi et al., 2015) which in turn strengthens their confidence in forward-looking career planning (Van Veldhoven et al., 2017). According to self-determination theory, when employees perceive a group-inclusive climate, they fulfill their internal psychological needs. With psychological security and commitment, employees are more inclined to make positive behavioral choices and invest in activities such as career planning and skill development beyond standard work requirements (De Vos & Soens, 2008).

**Hypothesis** **3.** *A group-inclusive climate has a positive impact on a salesperson’s career initiative.*

The satisfaction of internal needs drives employees to exhibit positive behaviors. According to social exchange theory, positive perceptions motivate employees to support their team in achieving objectives through reciprocal actions (Vadera et al., 2013). An employee’s sense of obligation acts as an internal catalyst, promoting long-term mutually beneficial relationships (Mossholder et al., 2005). Although prior studies by Yu and Frenkel (2013), Coyle-Shapiro et al. (2006) emphasize the role of felt obligation in enhancing task performance and service-oriented citizenship behavior, its potential to generate future outcomes remains underexplored. Felt obligation prompts employees to anticipate long-term reciprocal exchanges (Mossholder et al., 2005), which aligns with career initiative requirements such as self-motivation, proactivity, and persistence (Frese & Fay, 2001). Consequently, employees can construct long-term goal plans and obtain positive feedback for future work by improving their work status (Grant & Ashford, 2008).

**Hypothesis** **4.** *Felt obligation has a positive impact on the career initiative of salespeople.*

As discussed, we identified the correlation between team leaders’ perceptions and front-line employees’ positive behaviors. Based on social exchange theory, when supervisors perceive organizational support, they adjust their behavior to reciprocate with the team, thereby enhancing employees’ perception of support and fostering a climate of mutual respect and tolerance. According to self-determination theory, external environments and internal motivations jointly shape individuals’ autonomous behavioral choices. When salespeople perceive an inclusive team atmosphere, they develop greater trust in the working environment (Santuzzi et al., 2022) and experience psychological security (Lee & Shin, 2024). This fulfills their psychological needs, creating a sense of obligation for reciprocal exchanges. Moreover, employees’ desire for long-term social exchanges motivates them to engage in forward-looking professional initiative.

**Hypothesis** **5.** *SPOS promotes the salesperson’s felt obligation through a group-inclusive climate and ultimately stimulates the salesperson’s career initiative.*

### 2.3. Salesperson’s Core Self-Evaluation

Judge et al. (2003) argue that CSE reflects individuals’ most fundamental assessment, serving as a baseline for other beliefs and evaluations (Akosile & Ekemen, 2022). Ferris et al. (2011) found that high-CSE employees are more responsive to positive stimuli. In the workplace, they emphasize positive organizational stimuli, maintain a favorable view of the organization (Zhao et al., 2024), and exhibit stronger organizational identity (Yan et al., 2020) Furthermore, CSE significantly influences intrinsic motivation by fostering subjective initiative, self-efficacy, and positive psychological momentum, which encourages individuals to set challenging goals and cultivate perseverance (Gagné & Deci, 2005).

**Figure 1:**
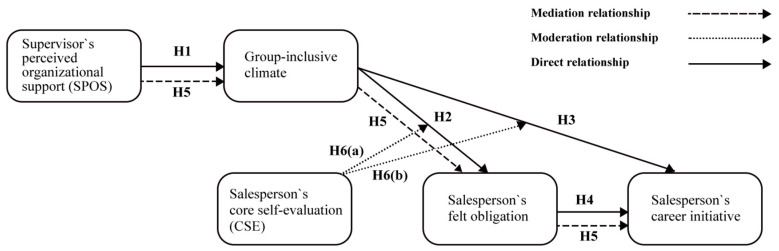
Research model.

In summary, CSE determines employees’ responsiveness to positive environmental stimulus and their ability to transform these into intrinsic motivation. High-CSE salespeople are more sensitive to environmental changes during self-determined decision-making, believing in their capacity to leverage external resources (Li & Ding, 2022). This belief enhances positive motivation and prompts feedback behaviors. Moreover, CSE determines whether employees possess the confidence to set challenging goals. Even in an inclusive environment, low self-awareness may hinder contributions to the organization. Consequently, CSE moderates the impact of a group-inclusive climate on felt obligation and career initiative. We put forward the following hypothesis. Moreover, Figure 1 depicts the interrelationships between the different variables investigated in this study.

**Hypothesis** **6(a).** *A salesperson’s CSE moderates the impact of a group-inclusive climate on their felt obligation. When a salesperson’s CSE is higher, the group-inclusive climate has a stronger impact.*

**Hypothesis** **6(b).** *A salesperson’s CSE moderates the impact of a group-inclusive climate on their career initiative. When a salesperson’s CSE is higher, the group-inclusive climate has a stronger impact.*

## 3. Materials and Methods

### 3.1. Research Approach

In this study, we employed a questionnaire survey method. First, this approach facilitates the collection and analysis of data (Hussain et al., 2023). Second, it enables the acquisition of reliable data from a substantial number of respondents within a constrained timeframe (Chaudhry et al., 2023). According to Hennessy and Patterson (Hennessy & Patterson, 2011), designing measurement instruments is a crucial initial step for conducting survey analysis. Consequently, we developed a measurement instrument to ensure effective data collection.

### 3.2. Instrument

The measurement instrument in this study encompasses five variables: SPOS, group-inclusive climate, felt obligation, career initiative, and CSE. We selected established scales from existing research that have been formally published and validated for effective measurement. To ensure cultural and linguistic accuracy, we conducted the translation and back-translation of the scales. Through a pilot test, we analyzed the reliability and validity of the scales and carried out revisions based on feedback from the respondents. Each scale achieved an acceptable Cronbach’s alpha value of 0.7 or higher. The measurement instrument consists of 41 items and is measured using a 5-point Likert scale (1 = “strongly disagree”; 5 = “strongly agree”). The specific dimensions are detailed below, and the complete scales are provided in Appendix A.

SPOS: We adopted the perceived organizational support scale developed by Eisenberger et al. (1986) to measure SPOS, which is widely utilized in organizational support research for employees (including supervisors) in enterprises and possesses high reliability and validity (Eisenberger et al., 2010, 2014, 2020). The scale comprises 8 items, including, “The organization places significant emphasis on my objectives and values” (Cronbach’s α = 0.76).

Group-inclusive climate: Our study adopts the measurement scale for group-inclusive climate developed by Chung et al. (2020), consisting of 10 total items, the first 5 of which are employed to measure employees’ sense of belonging to the team, including questions such as “I am treated as a valued member of my work group.” In contrast, the last 5 items are utilized to measure the extent to which employees perceive that the team is tolerant of their own uniqueness, including, “ I can offer a viewpoint on work-related matters that differs from those of my group members.” (Cronbach’s α = 0.73).

Felt obligation: The scale we used for felt obligation is that developed by Eisenberger et al. (2001). The 6-question scale encompasses items such as, “ I feel a personal responsibility to contribute in any way possible to assist my organization in reaching its objectives.” (Cronbach’s α = 0.72).

Career initiative: The scale adopted in our study to measure career initiative was developed by Van Veldhoven et al. (2017) and consists of 5 questions, including, “In my work, I keep trying to learn new things.” (Cronbach’s α = 0.78).

CSE: We adopted the CSE scale compiled by Judge et al. (2003), consisting of 12 items, of which 6 items are used for positive measurement and 6 for negative measurement. The contents of the scale include, “I am confident I will get the success I deserve in life.”, “Sometimes I feel depressed. (-).” (Cronbach’s α = 0.81).

### 3.3. Data Collection and Sampling

This study utilized a matched survey questionnaire method for data collection, wherein sales department supervisors and their sales staff completed questionnaires to evaluate different indicators. To minimize common method bias, the matched questionnaires were administered in three waves, with an interval of approximately one month between each collection. The first wave (T1) of data collection was conducted from May to June 2024, the second wave (T2) in August 2024, and the third wave (T3) in October 2024.

The enterprises investigated in this study are all located in Mainland China. Given our focus on the sales department, the size and nature of the enterprises were not considered as selection criteria. Initially, we employed convenience sampling, followed by snowball sampling to broaden the sample range. The electronic version of the questionnaire was sent to the sales team leaders, who were instructed to print it, complete it with their team members, and return the paper version to us. To ensure one-to-one correspondence between recipients across T1, T2, and T3 questionnaires, we collected the last four digits of the phone numbers of the team leaders and their employees from the T1 questionnaires.

A total of 50 teams participant completed all 3 waves of surveys, with 49 teams maintaining a supervisor-to-employee ratio of 1:6 and one team maintaining a ratio of 1:5. Overall, 50 supervisor questionnaires were successfully retrieved, achieving an effective retrieval rate of 83.3%. Additionally, 299 employee questionnaires were successfully retrieved, resulting in an effective retrieval rate of 83.1%. The detailed distribution process is described below:

Time 1: The T1 questionnaire comprised two sections: one for supervisors (variable: SPOS) and one for employees (variable: group-inclusive climate). A total of 60 supervisor questionnaires and 360 employee questionnaires were distributed, with each supervisor distributing 6 employee questionnaires. Ultimately, 52 supervisor questionnaires (retrieval rate: 86.7%) and 345 employee questionnaires (retrieval rate: 95.8%) were collected. Among the employee questionnaires, 312 were successfully matched with supervisor questionnaires, while 33 remained unmatched. The unmatched questionnaires were retained at T1 to allow for potential matching in subsequent surveys, as supervisors may complete their questionnaires later.

Time 2: The T2 questionnaire, completed by employees, included the variables “Felt Obligation” and “CSE”. We surveyed sales teams that had returned the T1 questionnaire (including unmatched responses). A total of 345 questionnaires were distributed, of which 310 were valid (effective rate: 89.9%). Among these, 300 questionnaires could be matched with T1 supervisor questionnaires, corresponding to 50 team supervisors. The remaining 10 questionnaires originated from two special cases: one team experienced a reduction in employee count from 6 to 5 due to turnover, while the other team’s supervisor supplemented the T1 questionnaire for a team of 5 employees.

Time 3: The T3 questionnaire, focused on the variable “Career Initiative”, was completed solely by supervisors who evaluated their respective sales teams. Based on T2 data, 52 teams were initially selected, and questionnaires were distributed accordingly. Ultimately, 50 questionnaires were successfully retrieved (retrieval rate: 96.2%). Data from two teams (one with 6 members and another with 5 members) were excluded due to non-response from their supervisors, resulting in the exclusion of data from 11 employees.

### 3.4. Statistical Methods

First, we provide a descriptive analysis of the demographics of supervisors and employees using frequency distributions and percentages.

Second, given the need to analyze multi-level data in this study, we employed the statistical power estimation method proposed by Scherbaum and Ferreter (2009) as well as the Monte Carlo simulation approach suggested by Arend and Schäfer (2019) to evaluate the adequacy of the sample size based on the collected data.

Third, to ensure that common method bias did affect the results, we conducted Harman’s single-factor test to examine whether the cumulative variance explained by the first unrotated factor exceeds 50% (Podsakoff & Organ, 1986). Furthermore, we introduced a common method latent factor into the structural equation model to assess potential changes in model fit indices, following the procedure outlined by Conway and Lance (2010).

Fourth, we utilized structural equation modeling (SEM) to perform confirmatory factor analysis (CFA) on the questionnaire. We constructed a five-factor model as the original hypothesized model, based on our research hypotheses, we combined the variables “SPOS” with “group-inclusive climate”, “group-inclusive climate” with “salesperson’s felt obligation”, “group-inclusive climate” with “salesperson’s career initiative”, and “salesperson’s felt obligation” with “salesperson’s career initiative” to develop four alternative four-factor competing models for comparison. Additionally, a two-level model was constructed to evaluate the structural validity of the questionnaire under cross-level conditions. Simultaneously, we calculated the average variance extracted (AVE) and the composite reliability (CR) of the questionnaire results to confirm its validity.

Finally, for hypothesis testing, we applied a multilevel structural equation modeling (MSEM) approach to analyze the nested data in this study and assess both the direct and indirect effects among the variables. Additionally, we tested the significance of the interaction terms on the outcome variable and visualized the interaction effects using graphical representations. Following the recommendations of Preacher et al. (2006), we further applied the Johnson-Newman technique to examine the practical significance of the conditional effects.

We utilized Mplus 8.0 software for data analysis, and the syntax was guided by the user’s guide provided by Muthén and Muthén (2017).

## 4. Results

### 4.1. Demographics

The final sample demographic data, after collection and organization, are presented below. The sample comprises 50 supervisors and 299 employees. The background information of effective questionnaire fillers for supervisors is shown in Table 1. Among the sample of managers, 68% were male (N = 34), and 94% had a college education or above (N = 47). Their ages were mainly between 26 and 45 years old (N = 38, 76%), and most teams had a total of 3–10 people (N = 42, 84.0%).

Table 2 shows the background information on employees. The proportion of male and female employees in the sample was relatively balanced (the male sample accounted for 50.2%), their ages were concentrated mainly under 35 years old (N = 266, 88.9%), 89.3% (N = 267) had been working in the team for less than 5 years, and those with a college degree or above accounted for 95.0% (N = 284).

### 4.2. Statistical Power

Based on the statistical power estimation method, with an ICC(1) value of 0.1 and a medium effect size of 0.5, the sample combination of six individuals per team and 50 teams achieves a statistical power of 0.94, exceeding the commonly accepted threshold of 0.8. Additionally, through Monte Carlo simulation, the statistical power obtained in this study also surpasses the threshold of 0.8.

### 4.3. Common Method Bias Check

We conducted Harman’s single-factor test, which revealed that the cumulative explained variance of the first unrotated factor was 30.22%, significantly lower than the acceptable threshold of 50%. 

Subsequently, we treated all measured items as indicators of a common method latent factor and constructed a structural equation model incorporating this latent factor. This modified model was then compared with the original model. The change in RMSEA was less than 0.005 (RMSEA = 0.043, ΔRMSEA = 0.001), and the changes in CFI and TLI were both less than 0.01 (CFI = 0.986, ΔCFI = 0.001; TLI = 0.981, ΔTLI = −0.001). Therefore, based on these results, there is no evidence of common method bias in this study. Therefore, this study does not exhibit common method bias.

### 4.4. Reliability and Validity Analysis

Table 3 presents discriminant validity results. The results indicate that the AVE values of all variables in this study are greater than 0.5, the composite reliability is greater than 0.8, and the correlation coefficients between all dimensions are less than the square roots of the AVE values; thus, the discriminant validity of our research is deemed adequate. Furthermore, all alpha coefficients exceed 0.8, indicating that the reliability of this questionnaire is outstanding.

Our five-factor model can better reflect the latent characteristics of common factor constructs (Table 4). Compared with other competing models, the content of the five-factor model is consistent (CFI = 0.985 > 0.9; TLI = 0.982 > 0.9; RMSEA = 0.042 < 0.05; SRMR = 0.031 < 0.05), meanwhile, the 2-level 5-factor model also demonstrates relatively high consistency (CFI = 0.988 > 0.9; TLI = 0.985 > 0.9; RMSEA = 0.05 < 0.05; SRMR (Within) = 0.027 < 0.05; SRMR (Between) = 0.020 < 0.05).

### 4.5. Hypothesis Testing

The test results of the direct and indirect effects of the model hypothesis are presented in Table 5 and Table 6, in which it can be observed from the direct effects that SPOS positively influences the group-inclusive climate (β = 0.251, *p* < 0.01). Hypothesis 1 is valid; group-inclusive climate can significantly affect salesperson’s felt obligation (β = 0.469, *p* < 0.001) and career initiative (β = 0.538, *p* < 0.001). Hypothesis 2 and Hypothesis 3 are also valid; salesperson’s felt obligation can promote their establishment of career initiative (β = 0.457, *p* < 0.001). Moreover, Hypothesis 4 is also valid.

The test results show that the chain mediation effect is significant. Group-inclusive climate and salesperson’s felt obligation play indirect roles in the relationship between SPOS and salesperson’s career initiative (β = 0.054, *p* < 0.01). Hypothesis 5 is thus proven.

**Figure 2:**
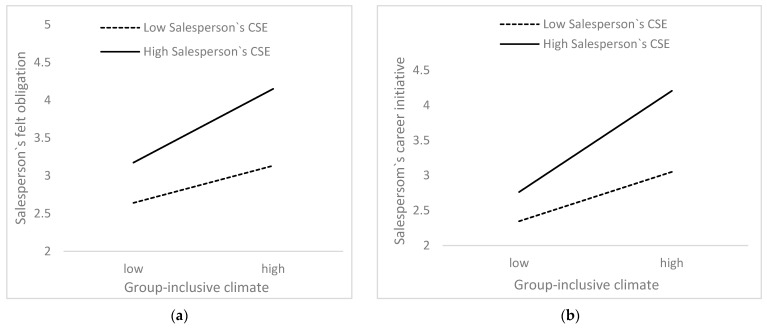
(**a**) Moderating effect of a salesperson’s CSE on the group-inclusive climate–salesperson’s felt obligation; (**b**) moderating effect of a salesperson’s CSE on the group-inclusive climate–salesperson’s career initiative.

Table 7 presents the positive moderating effect of a salesperson’s CSE. When salespeople with higher CSE perceive a group-inclusive climate, felt obligation (β = 0.176, *p* < 0.01) and career initiative (β = 0.270, *p* < 0.001) can be significantly enhanced; thus, Hypothesis 6(a) and Hypothesis 6(b) are valid. Figure 2 presents the interaction graphs with the mean of the regulating variable plus or minus one standard deviation.

**Figure 3:**
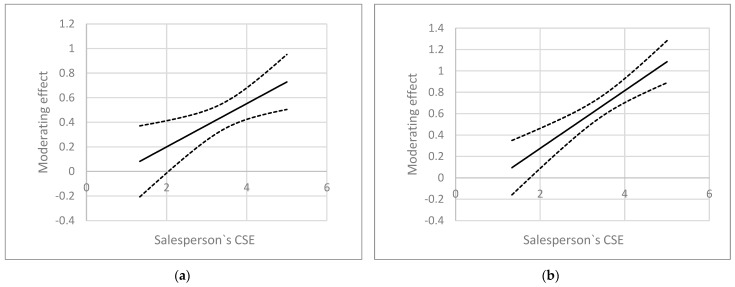
(**a**) Johnson–Newman figure of the moderating effect on salesperson’s felt obligation; (**b**) Johnson–Newman figure of the moderating effect on salesperson’s career initiative.

We adopted the Johnson–Newman technique for our study in order to explore the nature of the adjustment effect of Hypothesis 6. To facilitate observations and conclusions, we did not centralize the data, and the results are presented in Figure 3. The upper and lower two curves in the figures are 95% confidence intervals for indirect effects, and the middle curve represents the predicted values of moderating effects. When salesperson’s CSE is greater than 2.04, the indirect effect of the group-inclusive climate on the salesperson’s felt obligation increases; when salesperson’s CSE is greater than 1.76, the indirect influence of group-inclusive climate on salesperson’s career initiative also increases. These results also illustrate the significant role that a salesperson’s CSE plays in the relationship between their perception of the work environment and their reciprocal behavior.

### 4.6. Results Conclusions

After a systematic analysis, the research findings indicate that the sample size of this study possesses sufficient statistical power, exhibits no common method bias, and demonstrates satisfactory reliability and validity of the questionnaire. To present the hypothesis testing of this study more clearly, we have systematically summarized the results in Table 8.

## 5. Discussion

For salespeople, many performance-related incentives provided by enterprises not only fail to offer adequate motivation but also become a significant source of pressure (Lewin & Sager, 2009). However, research on how to stimulate proactive behavior among sales personnel remains limited, particularly from the perspective of front-line managers (Zhao et al., 2024). This study addresses this gap by constructing an influence chain from the organizational support perceived by front-line supervisors to the career proactiveness of front-line employees. It explores the sequential impact mechanism involving supervisor perception, organizational environment, employees’ psychological motivation, and their behavioral choices.

Firstly, SPOS can influence the formation of an inclusive climate within the team. The results of this study confirm H1. Prior research has demonstrated that SPOS enhances the perceived support for employees (Eisenberger et al., 2014; Pan et al., 2012), fostering a positive social exchange (Eisenberger et al., 2014). Employees view supervisors as organizational agents (Shanock & Eisenberger, 2006). When supervisors transmit the support they perceive from the organization, employees equate this support with that of the entire team. Consequently, a mutually supportive and cooperative team atmosphere is established, resulting in an inclusive climate that employees can perceive.

The confirmation of H2 and H3 demonstrates that an inclusive climate contributes to enhancing sales personnel’s felt obligation and career initiative. In conjunction with the validation of H4, when sales personnel perceive an inclusive external environment, positive self-selection (e.g., proactive behavior) reflects their psychological state of feeling obligated to reciprocate the organization, which aligns with the principles of self-determination theory. Consequently, we have identified a mechanism linking SPOS to the proactive behavior of front-line sales personnel, as evidenced by the results validating H5.

Although Hypothesis H5 has been supported, it is essential to differentiate the “group-inclusive climate,” which is central to this study, from other latent variables. This distinction is necessary because other factors may still significantly influence outcomes. Prior research has predominantly focused on LMX (e.g., Pan et al., 2012) or leadership style (e.g., Cheng et al., 2022), which primarily describe individual managerial behaviors rather than team-level dynamics. By contrast, group-inclusive climate, as a team-level construct, builds upon supervisors’ actions to foster an environment conducive to employee behavior improvement. While some scholars emphasize organizational-level variables such as team cohesion (Salloum et al., 2022) and team diversity (Tang et al., 2017), group-inclusive climate specifically highlights employees’ sense of belonging within the team. This sense of belonging serves as a key driver of intrinsic motivation (Shore et al., 2011), going beyond mere responses to external changes. Furthermore, front-line salespeople, who frequently work outside fixed locations (Lyngdoh et al., 2023), may have a weaker perception of “team.” In this context, fostering a sense of belonging becomes particularly critical. Consequently, group-inclusive climate provides a more precise framework for understanding the relationship between the team environment and the intrinsic motivation of front-line salespeople.

It is also important to note that the results indicate no significant direct effect of SPOS on employees’ sense of obligation (β = −0.030, *p* > 0.05) or career initiative (β = −0.033, *p* > 0.05). This suggests that the path from SPOS to sales personnel’s career initiative is fully mediated. First, given the complexity of chain mediation, potential latent variables may weaken the direct relationship between supervisor perception and employee proactive behaviors, and these influences cannot be entirely ruled out. Second, logically, the high autonomy of front-line sales personnel depends on both the work environment and their intrinsic motivation. Supervisor perception, as an internal attribute of supervisors, does not directly engage in employees’ self-determination process. Therefore, supervisors must enhance the work environment to ultimately influence employee behavior.

A high degree of decision-making autonomy is a critical component of the role attributes of front-line sales personnel (Boshoff & Allen, 2000). We consider a salesperson’s CSE as an intrinsic factor. The results of H6 verification indicate that CSE significantly moderates the positive impact of a group-inclusive climate. Specifically, when CSE is high, salespersons are more likely to exhibit positive psychological motivation to repay the organization and set more challenging personal goals. Additionally, this study finds that CSE has a significant direct effect on both felt obligation (β = 0.516, *p* < 0.001) and career initiative (β = 0.505, *p* < 0.001). This underscores the importance of individuals’ basic self-perception in shaping their responses to external environments and guiding self-determined behavior.

### 5.1. Theoretical Contribution

Despite the well-documented positive effects of proactive behavior on organizations, the role of front-line managers—those closest to employees—is often overlooked (Kou et al., 2022). This study investigates the relationship between front-line managers’ perceptions and their team members’ behavior, elucidating the influence pathway of organizational support. Prior research attributes front-line employees’ perception of organizational support primarily to the organization itself (Pan et al., 2012), neglecting specific recipients and transmission mechanisms. It is critical to examine how front-line managers’ perceptions shape both their own behavior and that of their team members. These perceptions affect employees’ views of job characteristics (Cropanzano & Mitchell, 2005), fostering emotional commitment and autonomous motivation, which drive proactive behavior (Ayed et al., 2024). Compared to studies on leadership styles or managerial personality traits, organizational support perceived by front-line managers can be systematically controlled and regulated by upper management, providing practical implications for business operations. By cultivating such an environment (Ding et al., 2022), front-line managers can enhance their attitudes and behaviors, positively influencing employees (Op De Beeck et al., 2018). This addresses the first research question.

Based on self-determination theory, we have elucidated the influence mechanism from front-line managers’ perceptions to the behavior of front-line employees. Previous studies provided only a general description of this mechanism (Zhao et al., 2024) and overlooked critical factors such as external environmental influences and employees’ intrinsic motivation (Deci et al., 2017). By incorporating group-inclusive climate and employees’ felt obligation, we bridge the self-determination chain between supervisors and employees, thereby addressing the second research question of this study.

Additionally, we place a higher emphasis on the way salespeople perceive the team environment since, compared with employees in other departments, salespeople have flexible personalized contracts regarding the workplace and working hours, which ensure their social participation and performance in the team (Kalra et al., 2024). It is necessary for us to undertake a more detailed analysis of the sales staff group. After all, the generation of proactive behavior not only depends on an individual’s perception of the external environment but also on their own personality traits (Zhao et al., 2024). We consider the intrinsic basis of a salesperson’s personality traits, CSE, as a key factor in addressing the third research question of this study. This extends prior research (e.g., Meng et al., 2023) on the influence of organizational support on employees’ proactive behaviors. The results demonstrate that the development of career initiatives does not solely depend on environmental changes; the role of CSE is also crucial. Perhaps initiative is not truly universal. Among people with lower CSE, the loss of self-efficacy hinders them from engaging in their work more enthusiastically, even when they are well-off.

### 5.2. Managerial Implications

The research findings of this study hold significant practical implications for enterprises. Firstly, motivating employee initiative remains a critical challenge for organizations (Van Veldhoven et al., 2017; Hirschi, 2014). In this context, we have re-evaluated the importance of front-line managers’ perceptions. Currently, enterprises often overlook the significance of front-line managers. Compared to other managerial levels, front-line managers face greater challenges and pressures (Huo et al., 2020). However, they receive limited benefits and are less likely to experience emotional or instrumental support from the organization (Karltun et al., 2023).

Therefore, organizations should implement targeted support measures for front-line managers and sales personnel. First, organizations can empower front-line managers by delegating authority, enabling them to independently design team incentive mechanisms. Front-line managers, being more familiar with market dynamics, can swiftly adjust team incentives and human resource allocation in response to market changes, thereby improving the efficiency of information feedback. Second, enterprises should enhance stress-resilience training for front-line managers and sales personnel to strengthen the overall stress tolerance of the sales team. Third, by establishing relevant awards, organizations can encourage managers to proactively refine team work strategies and develop personalized performance goals for front-line sales personnel. This facilitates the transfer of team support from managers to front-line sales personnel. Fourth, a more flexible evaluation mechanism should be implemented for front-line managers, allowing them to learn from mistakes during the management process and improve their skills. Finally, smaller units can be established within the sales department, providing front-line sales personnel with opportunities to rotate in managing small teams. Through practical experience, they can acquire management skills and foster a competition–cooperation dynamic among various small teams, ultimately enhancing the overall performance of the sales department.

Secondly, the findings of this study offer managers insights into motivating front-line salespeople. Beyond daily performance evaluations and salaries tied to work outcomes, managers should place greater emphasis on the contributions of front-line salespeople to the team, while addressing their work-related and family-related needs, thereby strengthening their sense of belonging within the team. Additionally, flexible work arrangements and idiosyncratic deal mechanisms function as effective tools for enhancing team belongingness, fostering self-identity, and cultivating felt obligation among salespeople. For salespeople who are less constrained by formal rules, recognition of their value by the organization and management is more likely to stimulate their initiative than rigid rules and regulations.

Additionally, it is necessary to screen the personality traits of salespersons as they need to consciously engage in sales work without supervision; thus, their CSE plays a crucial role in determining their sense of duty and initiative in work operations. Consequently, managers must thoroughly screen employees or assist them in establishing higher CSE levels through encouragement and support.

## 6. Conclusions, Limitations, and Future Research

### 6.1. Conclusions

This study posits that the organizational support perceived by front-line supervisors significantly influences the career initiative of grassroots salespeople. By focusing on the perspective of front-line supervisors, we have identified an effective pathway from supervisor perception to employee behavior, elucidating the antecedents of career initiative in front-line salespeople and reevaluating the critical role of front-line supervisors in organizations. Building on self-determination theory, we explored both external environmental factors and internal motivational drivers, constructing a comprehensive mechanism through which SPOS affects employee proactive behavior. The inclusive climate elucidates how supervisors can foster favorable interactive conditions for employees, offering a more nuanced understanding compared to traditional interpretations of organizational support. Felt obligation reflects employees’ willingness to reciprocate improvements in their work environment. Furthermore, this study examines the role of core self-evaluations, a fundamental personality trait, explaining why some employees exhibit higher levels of proactiveness despite receiving similar levels of organizational support. Overall, this research contributes to the literature on organizational support theory, offering new insights for scholars studying front-line managers and providing practical implications for enterprise management.

### 6.2. Limitations and Future Directions

Firstly, the limited sample size represents one of the primary limitations of this study. Although the surveyed enterprises were not restricted by scale, research on start-ups remains notably underrepresented. Compared to mature enterprises, start-ups typically adopt flatter organizational structures, which may attenuate the transmission of organizational support from supervisors to employees. This warrants further targeted investigation. Then, in a cross-cultural context, there are marked differences in the situations faced by grassroots managers and front-line sales personnel, both within and across organizations. Additionally, enterprises encounter diverse customer bases, with consumer-oriented and business-oriented sales exhibiting significant distinctions. Therefore, future research should incorporate more diverse and representative samples or focus on a more narrowly defined area to extend the research findings.

Secondly, the perceptions of the workplace environment among salespeople and among employees in other departments remain to be examined. Undoubtedly, a sales team possesses a particularity, exhibiting higher correlations between return and performance, yet the time and location of their work are more flexible, resulting in stronger or weaker perceptions within the sales team; this is an area that is not covered in this article, but it deserves more extensive and in-depth exploration.

Thirdly, based on the latest research, Shore and Chung (2024) propose that inclusivity is hierarchical, including tiers such as organizational, supervisor-perceived, and team member-perceived inclusivity, as well as factors such as group-inclusive climates, etc. Compared with the inclusivity perceived by supervisors and team members, a group-inclusive climate is more extensive and not confined to a specific group. Additionally, the manager is also a member of the team, and the group-inclusive climate also applies to the manager. In fact, a group-inclusive climate stems not only from the manager’s superior leadership or the overall company environment but also from the managerial team, which comprises colleagues and subordinates at the same level; therefore, the environment formed by the perception and behavior of a team manager needs to be evaluated at multiple levels. We may continue this discussion in the future by taking relevant perspectives (Shore & Chung, 2024; Chung et al., 2024) into account.

Finally, there is still a dearth of extensive research on the issue of inclusion failure. Inclusivity does not necessarily exert an influence on all employees with diverse characteristics, and it does not necessarily have a positive promoting effect in organizations with distinct characteristics. While this study takes employees’ CSE as a moderating variable in order to observe the promoting or inhibiting effect of personal characteristics on perceived inclusivity, there are still numerous factors that play such a role, such as power concentration at the team level, which remain to be explored. Similarly, as described by Zhao et al. (2024), the manager’s CSE is also a topic that is worthy of discussion; after all, even if managers perceive adequate organizational support, it is questionable whether they have the ability and determination to convey such support.

Despite these limitations, our study intends to stimulate the academic community in order to broaden its comprehension of the interactions of behavior and motivation between supervisors and employees; to assist managers in implementing interventions with the intent of enhancing work output; and to aid researchers who aspire to better understand the relationships between supervisors and employees.

## Figures and Tables

**Table 1:** Background information on supervisors.

Variables	Content	Quantity	Percentage
Gender	Male	34	68.0%
	Female	16	32.0%
Age	Under 25 years old	4	8.0%
	26–35 years old	26	52.0%
	36–45 years old	12	24.0%
	Age 46 and older	8	16.0%
Education	Junior high school or below	0	0.0%
	High School	3	6.0%
	Undergraduate student	42	84.0%
	Graduate student and above	5	10.0%
Time spent as a supervisor	1 year	3	6.0%
	2–5 years	33	66.0%
	More than 5 years	14	28.0%
Business size	Under 20 people	4	8.0%
	21–50 people	20	40.0%
	51–100 people	16	32.0%
	More than 100 people	10	20.0%
The size of the team managed	Less than 3 people	0	0.0%
	3–10 people	42	84.0%
	11–20 people	7	14.0%
	More than 20 people	1	2%

N = 50.

**Table 2:** Background information on employees.

Variables	Content	Quantity	Percentage
Gender	Male	150	50.2%
	Female	149	49.8%
Age	Under 25 years old	134	44.8%
	26–35 years old	132	44.1%
	36–45 years old	22	7.4%
	Age 46 and older	11	3.7%
Education	Junior high school or below	2	0.7%
	High School	13	4.3%
	Undergraduate student	263	88.0%
	Graduate students and above	21	7.0%
Time within a team	1–2 years	198	66.2%
	3–5 years	69	23.1%
	6–10 years	20	6.7%
	More than 10 years	12	4.0%
Time spent with a supervisor	1 year	125	41.8%
	2–5 years	148	49.5%
	More than 5 years	26	8.7%

N = 299.

**Table 3:** Discriminate validity and alpha coefficients.

Factor	SPOS	GIC	FO	CI	CSE	Mean	S.D.	C.R.	AVE	α
SPOS	(0.748) ^4^					3.80	0.69	0.910	0.560	0.887
GIC ^1^	0.213 ** ^5^	(0.782)				3.34	0.81	0.940	0.611	0.931
FO ^2^	0.092	0.419 **	(0.879)			3.28	0.95	0.953	0.773	0.941
CI ^3^	0.048	0.582 **	0.605 **	(0.928)		3.10	0.98	0.969	0.862	0.960
CSE	−0.029	0.070	0.431 **	0.434 **	(0.778)	3.43	0.84	0.948	0.605	0.940

N = 299; ** *p* < 0.01. ^1^ GIC: Group-inclusive climate; ^2^ FO: salesperson’s felt obligation; ^3^ CI: salesperson’s career initiative; ^4^ values in the diagonal represent the square roots of the average variance extracted values; ^5^ values in the area below the diagonal represent the correlation coefficients for the constructs.

**Table 4:** Confirmatory factor analysis.

Measurement Model	df	*χ* ^2^	*χ*^2^/df	CFI	TLI	RMSEA	SRMR
Hypothesized 5-factor model	160	245.4	1.53	0.985	0.982	0.042	0.031
M1 4-factor model (combined SPOS and GIC ^1^)	164	1474.917	8.99	0.772	0.736	0.164	0.210
M2 4-factor model (combined GIC and FO ^2^)	164	1100.270	6.71	0.837	0.811	0.138	0.119
M3 4-factor model (combined GIC and CI ^3^)	164	1078.341	6.58	0.841	0.816	0.137	0.108
M4 4-factor model (combined FO and CI)	164	924.844	5.64	0.868	0.847	0.125	0.068
2-level 5-factor model ^4^	100	159.617	1.60	0.988	0.985	0.045	0.027 (Within)
							0.020 (Between)

N = 299. ^1^ GIC: Group-inclusive climate; ^2^ FO: salesperson’s felt obligation; ^3^ CI: salesperson’s career initiative. ^4^ Level-2 factor: SPOS, Level-1 factors: group-inclusive climate, salesperson’s felt obligation, CSE and salesperson’s career initiative.

**Table 5:** Results of the direct effect analysis.

Direct Effect	Estimate	S.E.	95% C.I.
Between Level			
SPOS → GIC (Hypothesis 1) ^1^	0.251 **	0.089	(0.105, 0.397)
SPOS → FO ^2^	−0.030	0.073	(−0.150, 0.089)
SPOS → CI ^3^	−0.033	0.078	(−0.162, 0.095)
Within Level			
GIC → FO (Hypothesis 2)	0.469 ***	0.058	(0.374, 0.565)
GIC → CI (Hypothesis 3)	0.538 ***	0.074	(0.416, 0.660)
FO → CI (Hypothesis 4)	0.457 ***	0.057	(0.363, 0.551)

N = 299; ** *p* < 0.01; *** *p* < 0.001. ^1^ GIC: Group-inclusive climate; ^2^ FO: salesperson’s felt obligation; ^3^ CI: salesperson’s career initiative.

**Table 6:** Results of indirect effect analysis.

Indirect Effect	Estimate	S.E.	95% C.I.
SPOS → GIC ^1^ → FO ^2^	0.118 **	0.041	(0.051, 0.185)
SPOS → GIC → CI ^3^	0.135 **	0.050	(0.052, 0.218)
GIC → FO → CI	0.215 ***	0.036	(0.156, 0.274)
SPOS → GIC → FO → CI (Hypothesis 5)	0.054 **	0.020	(0.022, 0.086)

N = 299; ** *p* < 0.01; *** *p* < 0.001. ^1^ GIC: Group-inclusive climate; ^2^ FO: salesperson’s felt obligation; ^3^ CI: salesperson’s career initiative.

**Table 7:** Results of moderating effect analysis.

	Model 1			Model 2		
	FO ^2^			CI ^3^		
	Beta	S.E.	95% C.I.	Beta	S.E.	95% C.I.
GIC ^1^	0.451 ***	0.054	(0.361, 0.540)	0.660 ***	0.048	(0.582, 0.739)
CSE	0.460 ***	0.052	(0.373, 0.546)	0.466 ***	0.046	(0.390, 0.542)
GIC × CSE (Hypothesis 6)	0.176 **	0.064	(0.071, 0.281)	0.270 ***	0.056	(0.178, 0.363)

N = 299; ** *p* < 0.01; *** *p* < 0.001. ^1^ GIC: Group-inclusive climate; ^2^ FO: salesperson’s felt obligation; ^3^ CI: salesperson’s career initiative.

**Table 8:** Results of hypothesis testing.

	Hypothesis	Result
H1	SPOS has a positive impact on a group-inclusive climate.	Supported
H2	A group-inclusive climate has a positive impact on a salesperson’s felt obligation.	Supported
H3	A group-inclusive climate has a positive impact on a salesperson’s career initiative.	Supported
H4	Felt obligation has a positive impact on the career initiative of salespeople.	Supported
H5	SPOS promotes the salesperson’s felt obligation through a group-inclusive climate and ultimately stimulates the salesperson’s career initiative.	Supported
H6(a)	A salesperson’s CSE moderates the impact of a group-inclusive climate on their felt obligation. When a salesperson’s CSE is higher, the group-inclusive climate has a stronger impact.	Supported
H6(b)	A salesperson’s CSE moderates the impact of a group-inclusive climate on their career initiative. When a salesperson’s CSE is higher, the group-inclusive climate has a stronger impact.	Supported

## Data Availability

The data presented in this study are available on request from the first author.

## Appendix A

**Table A1:** Measurement items.

Variables	Source	Cronbach’s Alpha
Supervisor’s perceived organizational support	(Eisenberger et al., 1986)	0.76
1. The organization values my contribution to its well-being.		
2. The organization strongly considers my goals and values.		
3. The organization really cares about my well-being.		
4. The organization is willing to help me when I need a special favor.		
5. The organization shows very little concern for me. (-)		
6. The organization cares about my general satisfaction at work.		
7. The organization cares about my opinions.		
8. The organization takes pride in my accomplishments at work.		
Group-inclusive climate	(Chung et al., 2020)	0.73
1. I am treated as a valued member of my work group.		
2. I belong in my work group.		
3. I am connected to my work group.		
4. I believe that my work group is where I am meant to be.		
5. I feel that people really care about me in my work group.		
6. I can bring aspects of myself to this work group that others in the group don’t have in common with me.		
7. People in my work group listen to me even when my views are dissimilar.		
8. While at work, I am comfortable expressing opinions that diverge from my group.		
9. I can share a perspective on work issues that is different from my group members.		
10. When my group’s perspective becomes too narrow, I am able to bring up a new point of view.		
Salesperson’s felt obligation	(Eisenberger et al., 2001)	0.72
1. I feel a personal obligation to do whatever I can to help my organization achieve its goals.		
2. I owe it to the organization to give 100% of my energy to organization’s goals while I am at work.		
3. I have an obligation to the organization to ensure that I produce high-quality work.		
4. I owe it to the organization to do what I can to ensure that customers are well-served and satisfied.		
5. I would feel an obligation to take time from my personal schedule to help the organization if it needed my help		
6. I would feel guilty if I did not meet the organization’s performance standards		
Salesperson’s career initiative	(Van Veldhoven et al., 2017)	0.78
1. In my work, I set challenging goals.		
2. In my work, I keep trying to learn new things.		
3. With regard to my skills and knowledge, I see to it that I can cope with changes in my work.		
4. I think about how I can keep doing a good job in the future.		
5. In my work, I search for people from whom I can learn something		
Salesperson’s core self-evaluation	(Judge et al., 2003)	0.81
1. I am confident I will get the success I deserve in life.		
2. Sometimes I feel depressed. (-)		
3. When I try, I generally succeed.		
4. Sometimes when I fail I feel worthless. (-)		
5. I complete tasks successfully.		
6. Sometimes, I do not feel in control of my work. (-)		
7. Overall, I am satisfied with myself.		
8. I am filled with doubts about my competence. (-)		
9. I determine what will happen in my life.		
10. I do not feel in control of my success in my career. (-)		
11. I am capable of coping with most of my problems.		
12.There are times when things look pretty bleak and hope less to me. (-)		
